# Supplementary material for: Delayed lubricin injection improves cartilage repair tissue quality in an in vivo rabbit osteochondral defect model
Source: bioRxiv. 2025 Feb 8:2025.02.06.636825. Preprint. [Version 1] doi: 10.1101/2025.02.06.636825 (PMC11839081; doi:10.1101/2025.02.06.636825)

Supplementary Material

Table S1. Scoring criteria for the modified ICRS score

	Modified ICRS	Points	
**1**	**Degree of defect repair**	In level with surrounding tissue	4
		75% repair of defect depth	3
		50% repair of defect depth	2
		25% repair of defect depth	1
		0% repair of defect depth	0
**2**	**Integration to border zone**	Complete integration with surrounding cartilage	4
		Demarcating border < 1mm	3
		3/4 of graft integrated, 1/4 with a notable border > 1mm width	2
		1/2 of graft integrated, 1/2 with a notable border > 1mm width	1
		From no contact to 1/4 of graft integrated with surrounding cartilage	0
**3**	**Macroscopic appearance**	Intact smooth surface	4
		Fibrillated surface	3
		Small, scattered fissures or cracks	2
		Several, small or few but large fissures	1
		Total degeneration of grafted area	0
**4**	**Color of repair tissue**	Hyaline, pearly like	4
		Predominantly hyaline, pearly like (>50%)	3
		Predominantly white (>50%)	2
		White	1
		No repair tissue	0
**Overall Repair Assessment**		Grade I	16
		Grade II	12 - 15
		Grade III	8 - 11
		Grade IV	4 - 7
		Grade V	0 - 3

Table S2. Scoring criteria for Goebel score

	Goebel	Points	

1	**Color of the repair tissue**	Hyaline, pearly like	4
		Predominantly hyaline, pearly like (>50%)	3
		Predominantly white (>50%)	2
		White	1
		No repair tissue	0
2	**Presence of blood vessels in the repair tissue**	No blood vessels	4
		Less than 25% of the repair tissue	3
		25-50% of the repair tissue	2
		50-75% of the repair tissue	1
		More than 75% of the repair tissue	0
3	**Surface of the repair tissue**	Smooth, homogeneous	4
		Smooth, heterogeneous	3
		Fibrillated surface	2
		Incomplete new repair tissue	1
		No repair tissue	0
4	**Filling of the defect**	In level with adjacent cartilage	4
		>50% repair of defect depth or hypertrophy	3
		<50% repair of defect depth	2
		0% repair of defect depth	1
		Subchondral bone damage	0
5	**Degeneration of adjacent articular cartilage**	Normal	4
		Cracks and/or fibrillations in integration zone	3
		Diffuse osteoarthritic changes	2
		Extension of the defect into the adjacent cartilage	1
		Subchondral bone damage	0
**Total points**			20

Table S3. Histopathological scoring criteria

	Parameter	Qualifications (%)	Points
1	**Defect Fill**	100	4
		75 - 99	3
		50 - 74	2
		25 - 49	1
		0 - 24	0
2	**Cell Distribution**	All	4
		67 - 99	3
		34 - 66	2

		1 - 33	1
		None	0
**3**	**Perilesional cloning**	None	3
		Seldom	2
		Occasional	1
		Frequent	0
**4**	**Lesion-perilesion tissue integration**	Complete	2
		Gap on one side	1
		Gap on both sides	0
**5**	**Subchondral bone attachment**	100	4
		75 - 99	3
		50 - 74	2
		25 - 49	1
		0 - 24	0
**6**	**Surface fibrillation**	None	3
		Slight fibrillation	2
		Moderate fibrillation	1
		Severe fibrillation	0
**7**	**tidemark reformation**	Complete	3
		51 - 99	2
		1 - 50	1
		None	0
**8**	**Saf-O staining**	91 - 100	4
		76 - 90	3
		51 - 75	2
		26 - 50	1
		0 - 25	0

Table S4. Subscores for modified ICRS gross scoring system. Means ± SD are reported.

Modified ICRS Subscore Parameter	Control (n=8)	Saline (n=6)	rhLubricin (n=6)
**Degree of Defect Repair**	4±0	2.8±0.8	3.1±0.4
**Integration to Border Zone**	4±0	2.2±0.8	2.6±0.4
**Macroscopic Appearance**	4±0	1.8±0.4	2.3±0.6
**Color of Repair Tissue**	4±0	2.3±0.3	2.4±0.7

Table S5. Subscores for Goebel gross scoring system. Mean ± SD are reported.

Goebel Subscore Parameter	Control (n=8)	Saline (n=6)	rhLubricin (n=6)
**Color of Repair Tissue**	4±0	2.3±0.3	2.4±0.7
**Presence of Blood Vessels**	4±0	2.1±1.4	2.8±0.6
**Surface of Repair Tissue**	4±0	2.1±0.2	2.3±0.6
**Filling of Defect**	4±0	2.8±0.6	3.3±0.3
**Degeneration of Adjacent Cartilage**	4±0	2.4±0.6	2.8±0.4

Figure S1. Safranin-O sections for all samples used in histological assessments (n=3/group). Scale bars = 1 mm.

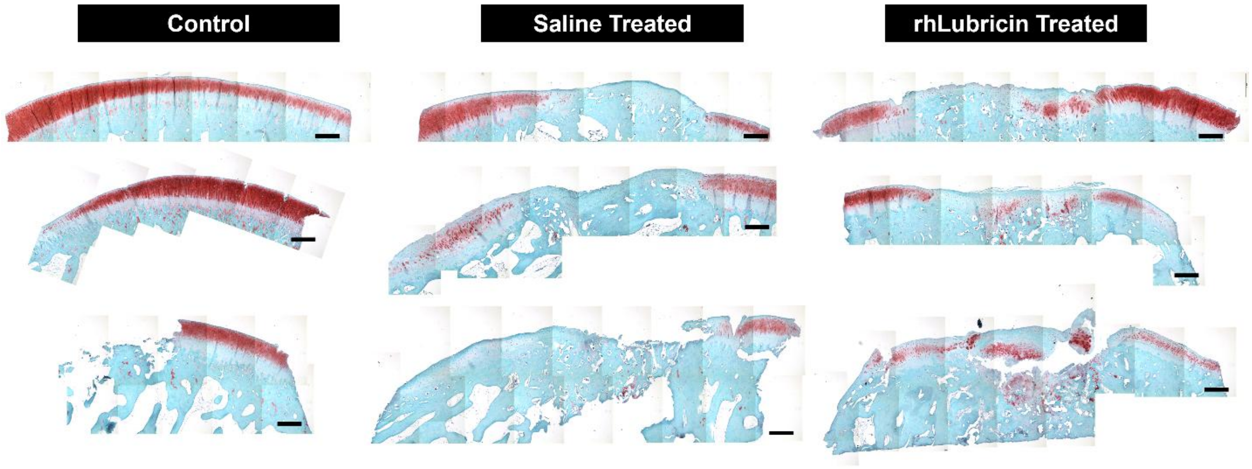

Supplement: Supplement 1 [file media-1.pdf]
